# Stimulatory Effect of Magnetite Nanoparticles on a Highly Enriched Butyrate-Oxidizing Consortium

**DOI:** 10.3389/fmicb.2018.01480

**Published:** 2018-07-05

**Authors:** Li Fu, Tianze Song, Wei Zhang, Jie Zhang, Yahai Lu

**Affiliations:** ^1^College of Urban and Environmental Sciences, Peking University, Beijing, China; ^2^College of Resources and Environment, Fujian Agriculture and Forestry University, Fuzhou, China

**Keywords:** syntrophy, butyrate, direct interspecies electron transfer, methanogenesis, wetland, Tibetan Plateau

## Abstract

Syntrophic oxidation of butyrate is catabolized by a few bacteria specialists in the presence of methanogens. In the present study, a highly enriched butyrate-oxidizing consortium was obtained from a wetland sediment in Tibetan Plateau. During continuous transfers of the enrichment, the addition of magnetite nanoparticles (nanoFe_3_O_4_) consistently enhanced butyrate oxidation and CH_4_ production. Molecular analysis revealed that all bacterial sequences from the consortium belonged to *Syntrophomonas* with the closest relative of *Syntrophomonas wolfei* and 96% of the archaeal sequences were related to *Methanobacteria* with the remaining sequences to *Methanocella*. Addition of graphite and carbon nanotubes for a replacement of nanoFe_3_O_4_ caused the similar stimulatory effect. Silica coating of nanoFe_3_O_4_ surface, however, completely eliminated the stimulatory effect. The control experiment with axenic cultivation of a *Syntrophomonas* strain and two methanogen strains showed no effect by nanoFe_3_O_4_. Together, the results in the present study support that syntrophic oxidation of butyrate is likely facilitated by direct interspecies electron transfer in the presence of conductive nanomaterials.

## Introduction

Methane is an end product of anaerobic food web degrading organic substances in anoxic habitats. Under methanogenic conditions where the electron donors other than protons and CO_2_ are absent, the complicated organic substances undergo fermentation producing short-chain alcohols and fatty acids as intermediate products (Drake et al., 2009). Secondary fermenters metabolize these products by discharging electrons to protons forming H_2_ or formate, which are then used by methanogens. This syntrophic cooperation, however, confronts a critical energetic dilemma as the syntrophic bacteria require a sufficiently low concentration of H_2_ or formate to process electron discharging that is in disfavor of methanogens (Schink, 1997; McInerney et al., 2009; Stams and Plugge, 2009; Schink et al., 2017).

The theory of syntrophic methanogenesis was discovered a half century ago (Bryant et al., 1967). The pioneering work considered H_2_ as mediator for interspecies electron transfer. Later, formate was found to serve as a similar function (Thiele and Zeikus, 1988; Boone et al., 1989; Dong and Stams, 1995). Using of formate can confer a kinetic advantage due to its faster diffusion rate than H_2_ in aqueous medium (Boone et al., 1989; Worm et al., 2014). Recently, amino acids like alanine was found to serve as a supplemental carrier for interspecies electron transfer (Walker et al., 2012).Amino acid exchange may not only function for electron transfer but become essential as some syntrophic partners evolve into amino acid auxotrophies over the course of syntrophic cooperation (Embree et al., 2015).

Apart from the mechanisms above, direct interspecies electron transfer (DIET) has been revealed. This process was initially demonstrated in the coculture of two *Geobacter* species (Summers et al., 2010). Then it was found DIET also occurred between *Geobacter* and methanogens with ethanol as the sole substrate (Morita et al., 2011). *Methanosarcina barkeri* and *Methanosaeta harundinacea* seemed particularly efficient in performing DIET with *Geobacter* (Rotaru et al., 2014a,b). Strikingly, addition of the electrically conductive granular activated carbon facilitated DIET either between *Geobacter* species or between *Geobacter* and *Methanosarcina* (Liu et al., 2012). In paddy soil enrichments dominated by *Geobacter* and *Methanosarcina*, the production of CH_4_ from ethanol was significantly stimulated in the presence of magnetite nanoparticles (nanoFe_3_O_4_) (Kato et al., 2012a). Other conductive materials like biochar, graphite, and carbon cloth were found to promote DIET between *Geobacter* species and between *Geobacter* and methanogens (Chen et al., 2014a,b; Zhao et al., 2015a,b). DIET could also occur between *Geobacter* and nitrate reducer in the presence of conductive nanoFe_3_O_4_ (Kato et al., 2012b). Most of these studies, however, used ethanol (occasionally acetate) as substrate with *Geobacter* as syntrophic bacteria. *Geobacter* spp. are known to synthesize metallic-like conductive structure or e-pili and outer membrane *c* type cytochromes for electric conductivity (Lovley and Malvankar, 2015; Malvankar et al., 2015; Rotaru et al., 2015; Lovley, 2017).

Butyrate is a major intermediate during the decomposition of organic residues in anoxic environments. The syntrophic oxidation of butyrate is thermodynamically stricter than ethanol, requiring a much lower H_2_ partial pressure for the reaction to process (Schink, 1997; Schink et al., 2017). So far, only a few bacteria specialists are found to metabolize butyrate oxidation through obligate syntrophy with methanogens (Sieber et al., 2010, 2012, 2015). The pure cultures of butyrate syntrophs known to date do not contain genomic machinery for e-pili or outer membrane cytochromes found in *Geobacter* (Sieber et al., 2012, 2014). This information suggests that DIET shall not exist in butyrate oxidation. But the possibility cannot be ruled out if electric connection substitute is provided externally. Such a substitute has in fact been demonstrated in *Geobacter*-based cocultures. In a coculture of *Methanosarcina barkeri* with a pilin-deficient *Geobacter metallireducens* the addition of granular activated carbon restored the otherwise broken DIET activity (Rotaru et al., 2014a). It has also been suggested that nanoFe_3_O_4_ can complement the function of pilin-associated *c* type cytochrome OmcS for DIET in *Geobacter sulfurreducens* (Liu et al., 2015).

Two kinds of studies with conflict results have been reported on butyrate syntrophy. On the one hand, the studies with environmental enrichments proposed that DIET likely occurred for butyrate oxidation in the presence of the conductive nanoFe_3_O_4_ (Li et al., 2015; Zhang and Lu, 2016). On the other hand, the study on the defined coculture showed that the addition of conductive carbon nanotubes (CNTs) stimulated not only the coculture comprising *Syntrophomonas wolfei* and *Methanospirillum hungatei* but also some pure culture methanogens (Salvador et al., 2017). Robust conclusions, however, are difficult to obtain from these studies. In case of environment enrichments, microbial compositions were too complicated to tease out explicitly the routes of interspecies electron transfer (Li et al., 2015; Zhang and Lu, 2016). In case of the defined coculture, the influence of CNTs varied depending on methanogen identity (Salvador et al., 2017). Apparently, more researches are needed to evaluate the possibility of DIET in butyrate syntrophy.

In the present study we constructed a highly enriched cultivation from a wetland soil collected from Zoige wetland in Tibetan Plateau. The enrichment was dominated exclusively by *Syntrophomonas* and *Methanobacteria* without the detection of *Geobacter* and other bacteria. We found that addition of nanoFe_3_O_4_ and CNTs to the enrichment substantially accelerated syntrophic oxidation of butyrate while the test on a few pure cultures revealed no effect.

## Materials and Methods

### Soil Sampling

The surface soil samples were collected from an open fen close to the Wetland National Nature Reserve of Zoige located in Qinghai-Tibetan Plateau (33°47′ N, 102°57′ E) (Fu et al., 2015). The Zoige wetland covers a total area of 6,180 km^2^, with the average altitude of 3,500 m and the mean annual air temperature of around 1°C (Fu et al., 2015). Vegetation and organic debris was removed by hands during the sampling. Ten kilograms of wet soil samples were collected, placed in an ice box and transported to the laboratory within 24 h for immediate processing. The soil sample had the following characteristics: pH 7.5, organic C of 15.26 g kg^-1^, total N of 1.06 g kg^-1^, and C:N of 14.4. Soil slurries were prepared by mixing soil samples with autoclaved and degassed water. The slurries were passed through 2-mm sieves to homogenize and remove the coarse materials. Thirty grams of soil slurry was then filled into 50-ml glass bottles with the final soil (d.w.) to water ratio of 1:5. The bottles were closed with butyl stoppers and flushed with N_2_. Soil slurries were pre-incubated for 21 days at 30°C to reduce electron acceptors prior to the enrichment incubation.

### Enrichment Cultivation, Isotope Labeling, and Molecular Analysis

Enrichment incubation was initiated by inoculating 4% (v/v) pre-incubated soil slurry into 60-ml vessels containing 25 ml of Hepes-buffered (30 mM, pH 7.0) fresh medium under a headspace of N_2_/CO_2_ (80/20) (Supplementary Figure S4). The basal medium contained MgCl_2_.6H_2_O (0.4 g l^-1^), CaCl_2_.H_2_O (0.1 g l^-1^), NH_4_Cl (0.1 g l^-1^), KH_2_PO_4_ (0.2 g l^-1^), KCl (0.5 g l^-1^), and was supplemented with, vitamin and trace element solutions as described previously (Lü and Lu, 2012). Na_2_S.9H_2_O (1.0 mM) was applied to the growth medium together with redox indicator resazurin (0.0005 g l^-1^). Butyrate was added to a final concentration of 5 mM in the initial four transfers and then increased to 10 mM thereafter. Cysteine was not added to avoid the possible effect of electron shuttle molecules. Magnetite nanoparticles were synthesized as described previously (Kang et al., 1996). Graphite and multi-walled CNTs was purchased from Beijing Dk Nano Technology, China.

Continuous transfers were conducted in the presence of nanoFe_3_O_4_ (4.64 mM in Fe in the medium). The inocula for every transfer were taken from the last nanoFe_3_O_4_-amended cultivation. For a comparison, same inocula were used to make parallel preparations without nanoFe_3_O_4_ in the medium (i.e., the control) (Supplementary Figure S4).

The final cultivation (after 13 transfers) was used to extract microbial DNA following the previous protocol (Ma et al., 2012). DNA samples from both the control and nanoFe_3_O_4_ treatment were used to construct bacterial and archaeal clone libraries (Ma et al., 2012). The PCR amplification, cloning, and sequencing followed the previous procedure (Peng et al., 2008; Rui et al., 2009). Phylogenetic trees were constructed using the neighbor-joining algorithm of the MEGA7 program (Kumar et al., 2016), and bootstrap analysis implemented 1,000 replicates. DNA-SIP was performed using the same cultivation. For this purpose, the fully ^13^C-labeled butyrate (99 atom%; Sigma-Aldrich) was added as substrate. At the end of incubation, the carbon isotopic ratios (δ^13^C values) of CH_4_ and CO_2_ were analyzed by a gas chromatography-isotope ratio mass spectrometry system (Yuan and Lu, 2009). DNA was extracted from the ^13^C-labeled and non-labeled cultivations and subjected to DNA-SIP procedure through the isopycnic centrifugation and density gradient fractionation of DNA as described previously (Liu et al., 2011; Rui et al., 2011; Gan et al., 2012). The density-resolved DNA gradients were quantified for total bacteria and archaea using real-time quantitative PCR (Qiu et al., 2008; Gan et al., 2012). The fingerprinting of the DNA gradients was conducted using the terminal restriction fragment length polymorphism analysis (T-RFLP) following the protocol described previously (Liu et al., 2011; Rui et al., 2011; Gan et al., 2012).

### Tests on the Enrichment

The following materials were used for test on the enriched cultivation: (1) nanoFe_3_O_4_; (2) nanoFe_3_O_4_ coated with silica prepared as described (Deng et al., 2008; Li et al., 2015); (3) graphite; and (4) CNTs. For the test with CNTs, three further transfers were made. The inocula from the nanoFe_3_O_4_ treatment were used to prepare the first transfer (CNT-1) with the addition of 0.4% CNTs (w/v) in the medium. The second transfer (CNT-2) used the inocula from the CNT-1 treatment and incubated with the addition of CNTs or nanoFe_3_O_4_ in the medium. The third transfer was prepared to test the effect of CNTs concentration (0.2, 0.4, and 0.8%, w/v) (Supplementary Figure S4).

### Tests on Pure Cultures

Three organisms were used for pure culture test. *Methanocella conradii* (DSM 24694) was isolated and available in our lab (Lü and Lu, 2012). *Methanococcus maripaludis* (DSM 14266) were purchased from German culture collection DSMZ (Braunschweig, Germany). *Syntrophomonas erecta* subsp. *sporosyntropha* (DSM 16215) was a courtesy of Prof. Xiuzhu Dong at the Institute of Microbiology, Chinese Academy of Sciences. The *Methanocella conradii* was cultivated as described (Lü and Lu, 2012). The *Methanococcus maripaludis* was grown on 170 kPa of H_2_/CO_2_ (80:20, v/v) in a modified DSMZ141 medium containing 100 mM NaCl, 7.87 mM MgCl_2_.6H_2_O, and 0.007 mM Fe(NH_4_)_2_(SO_4_)_2_. The *Syntrophomonas erecta* was cultivated in medium containing 20 mM sodium crotonate as described previously (Zehnder and Wuhrmann, 1977; Wu et al., 2006). The effect of nanoFe_3_O_4_ was tested for these pure culture strains.

### Chemical Analyses

Gas samples (0.1 ml) were regularly taken from headspace of incubations with a pressure-lock precision analytical syringe (Baton Rouge, LA, United States). The concentrations of CH_4_ and CO_2_ were analyzed using gas chromatographs GC-7890 (Agilent Technologies, United States) equipped with a thermal conductivity detector (Li et al., 2015). Liquid samples (0.5 ml) were taken with sterile syringes and centrifuged 15 min at 17,949 ×*g* at 4°C. The supernatant was collected, passed through 0.25-μm-pore-size filters, and analyzed for the concentrations of acetate and butyrate with an HPLC-1200 using a Zorbax SB-AQ C18 column (Agilent Technologies, United States) (Zhang et al., 2014).

### Nucleotide Sequence Accession Numbers

The sequences of the 16S rRNA clones obtained in this study have been deposited in the EMBL nucleotide sequence database under the following accession numbers: KT203965–KT204256.

### Statistical Analysis

Data analyses were carried out using SPSS 16.0 (SPSS Inc., Chicago, IL, United States) software. For all the analyses, the significance level was set at *P* < 0.05. Sample variability is given as the standard (S.D.) of the mean.

## Results

**FIGURE 1:**
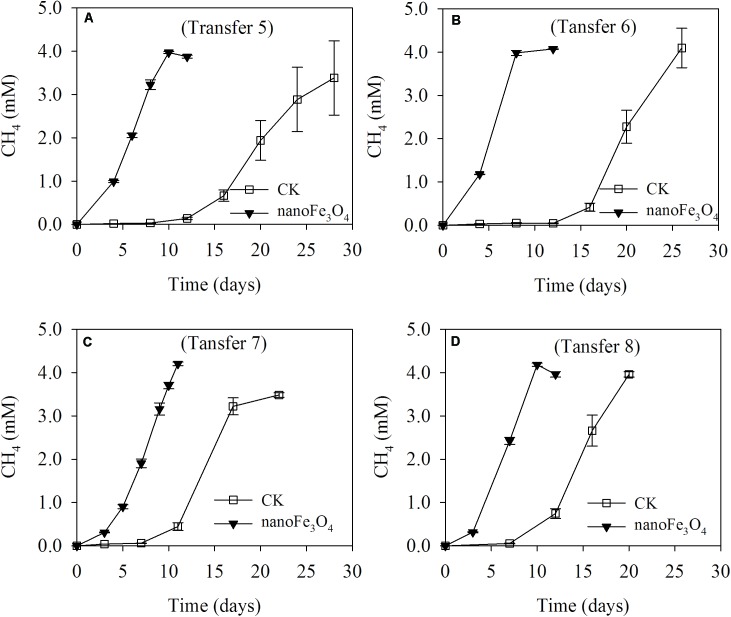
Methane production from butyrate oxidation in continuous (the 5th to 8th) transfers **(A–D)** of wetland enrichment in the presence (nanoFe_3_O_4_) and absence (CK) of nanoFe_3_O_4_. The concentration of CH_4_ produced was expressed as micromoles per liter (mM) of incubation medium. The error bars indicate the standard deviations of three replications.

The production of CH_4_ occurred without a lag in the initial two transfers indicating the readily activity of butyrate oxidation in this wetland sediment (Supplementary Figures S1A,B). Addition of 5 mM butyrate yielded about 10–12 mM CH_4_ (normalized to liquid volume) in the initial two transfers but then decreased to 2–2.5 mM CH_4_ in the third and fourth transfers (Supplementary Figures S1C,D). When the concentration of butyrate was increased to 10 mM in the later transfers, about 4 mM of CH_4_ was obtained (**Figure 1**). These results indicated that butyrate was stoichiometrically converted to CH_4_ and CO_2_ in the first two transfers, while thereafter the aceticlastic methanogens were lost (see more results below, **Figures 3A,C**) and CH_4_ was only produced from CO_2_ reduction by the electrons released from butyrate oxidation (four electrons per butyrate). After this transition, the addition of nanoFe_3_O_4_ consistently stimulated CH_4_ production, with the shorter lag phase and greater maximal rate compared with the control (**Figures 1A,B** and Supplementary Figures S1C,D). At the third transfer (right after the transition), CH_4_ production displayed a long lag in the control while it took less than a week before the onset of rapid production in the presence of nanoFe_3_O_4_ (Supplementary Figures S1C,D).

**FIGURE 2:**
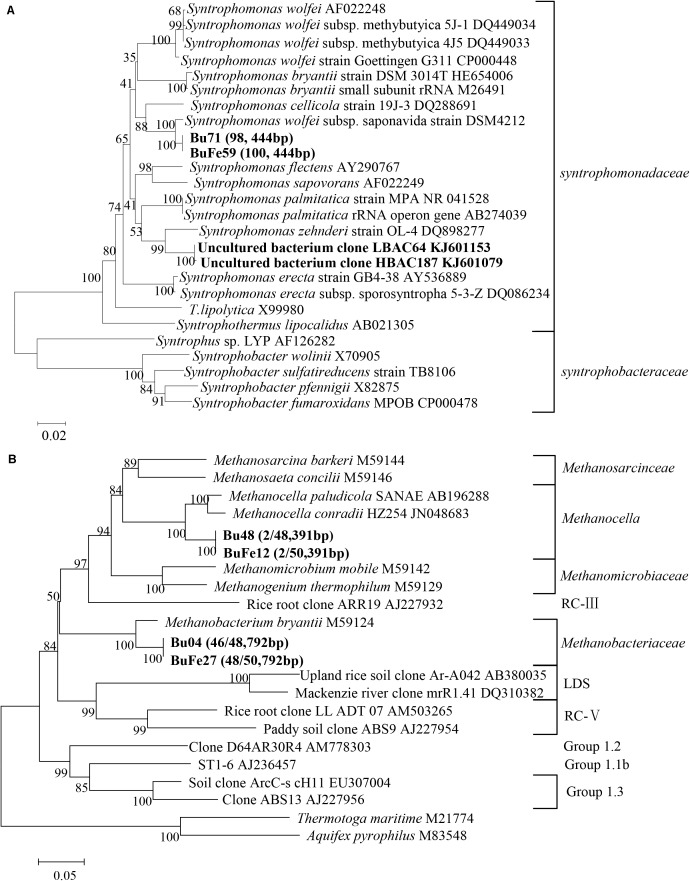
Phylogenetic relationship of representative bacterial **(A)** and archaeal **(B)** 16S rRNA gene clone sequences. Two bacterial and two archaeal clone libraries were constructed from nanoFe_3_O_4_ (BuFe) and the control (Bu) treatment, respectively. Sequences from these libraries were used for constructing the phylogenetic tree. Bootstrap values (%) were generated from 1,000 replications and indicated at individual nodes. The scale bar represents 10% sequence divergence. GenBank accession numbers of the reference sequences are indicated and *in silico* T-RF sizes are given in parentheses.

DNA-SIP and clone sequence analyses were used to determine microbial composition in the enriched cultivation. Prior to DNA-SIP, two bacterial and two archaeal clone libraries were constructed, with one each for the control and nanoFe_3_O_4_ treatment, respectively. All of the bacterial clone sequences from both the nanoFe_3_O_4_ treatment and the control were closely related to a *Syntrophomonas wolfei* strain (**Figure 2A**). For the archaeal composition, 94 out of 98 sequences were affiliated to *Methanobacteriales* (*Methanobacterium bryantii* as the closest pure culture relative) and the remaining four clones to *Methanocellales* (**Figure 2B**).

Albeit the simplicity of microbial composition in the enrichment, DNA-SIP was still performed by applying the fully ^13^C-labeled butyrate. Almost identical pattern was observed for the distribution of the density-resolved DNA fragments along the buoyant density gradient between the nanoFe_3_O_4_ treatment (Supplementary Figures S2C,D) and the control (Supplementary Figures S2A,B). Notably, the distribution of the archaeal DNA showed no difference between the labeled samples and the non-labeled controls (Supplementary Figures S2A,C), but the distribution of the bacterial DNA shifted to the heavier fractions in the labeled samples compared with the non-labeled control (Supplementary Figures S2B,D). These results indicate that the bacterial populations assimilated ^13^C from butyrate (regardless of nanoFe_3_O_4_ presence), while the archaeal populations did not. The T-RFLP fingerprinting of the density-resolved DNA gradients revealed only one peak for both archaeal and bacterial populations without difference between “heavy” and “light” DNA and between the nanoFe_3_O_4_ treatment (Supplementary Figures S3C,D) and the control (Supplementary Figures S3A,B). The T-RF had a size of 792 bp for the archaeal and 444 bp for the bacterial populations, respectively. Comparison to the clone sequences indicates that these T-RFs belong to *Methanobacteria* spp. and *Syntrophomonas* spp., respectively. Collectively, the molecular analyses indicate that the enrichment was almost close to be “purified.”

**FIGURE 3:**
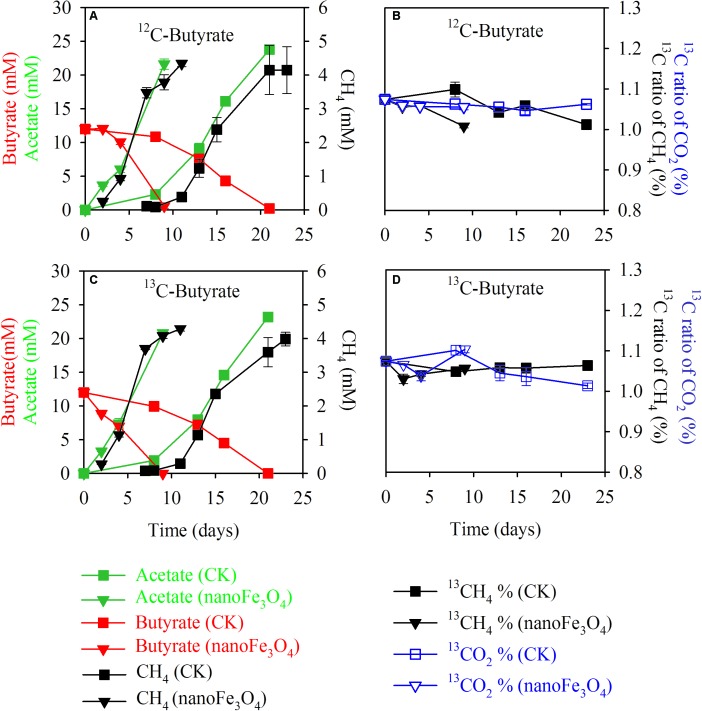
Isotopic labeling experiment in the 13th transfer enrichment. The fully ^13^C-labeled butyrate was applied for the labeling treatment. Shown are the total concentrations of CH_4_, butyrate, acetate **(A**,**C)** and the atomic ^13^C percentages of CH_4_ and CO_2_ **(B**,**D)** for the ^13^C-labeling treatment **(C**,**D)** and the non-labeling control **(A**,**B)**; with nanoFe_3_O_4_ (*inverted triangles*) or without nanoFe_3_O_4_ (*squares*). The total concentrations of acetate, butyrate, and CH_4_ were colored in *green*, *red*, and *black*, respectively **(A**,**C)**. The atomic ^13^C% of CH_4_ and CO_2_ were colored in *black* and *blue*, respectively **(B**,**D)**. The error bars indicate the standard deviations of three replications.

Isotopic and chemical analysis was conducted during the labeling experiment. Incubations with or without labeling showed identical patterns of butyrate consumption, CH_4_ production and acetate accumulation (**Figures 3A,B** without and **Figures 3C,D** with isotopic labeling). In consistence with the transfer incubations, addition of 10 mM butyrate produced about 4.0–4.3 mM CH_4_ and about 21–24 mM acetate cumulated in the medium (**Figure 3**). The ^13^C to ^12^C ratios of CH_4_ and CO_2_ showed no change over the course of incubations, being around 1.0–1.1% of atomic ^13^C for both CH_4_ and CO_2_ (**Figures 3B,D**) and there was no difference between the ^13^C labeled incubations (**Figure 3D**) and the non-labeled control (**Figure 3B**). These results indicate that CH_4_ was not labeled albeit the application of the fully ^13^C-labeled butyrate. Therefore, methanogens utilized only electrons but not carbon from butyrate oxidation.

**FIGURE 4:**
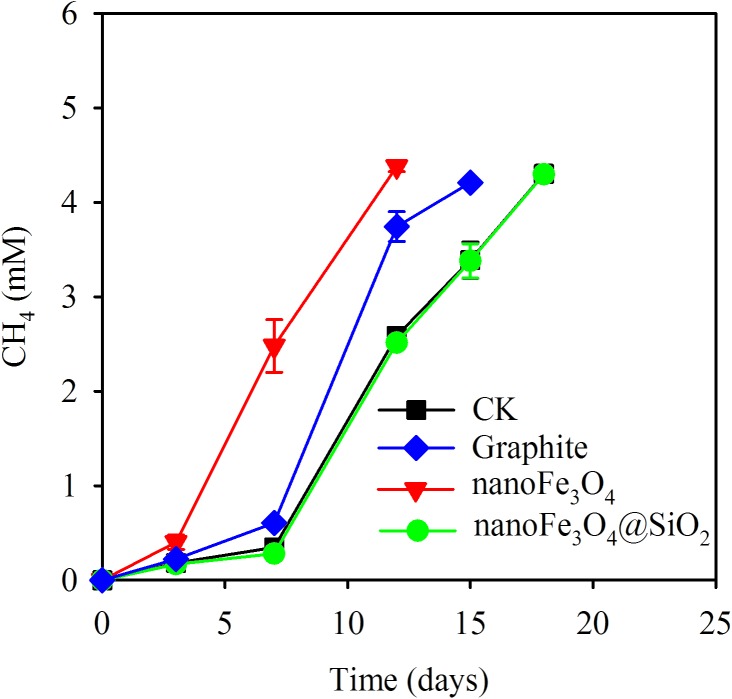
Methane production from butyrate oxidation in the 13th transfer of wetland enrichment. Treatments included: (i) addition of nanoFe_3_O_4_ (nanoFe_3_O_4_); (ii) addition of nanoFe_3_O_4_ coated with silica (nanoFe_3_O_4_@SiO_2_); (iii) addition of graphite nanoparticles (Graphite); and (iv) the control without nanomaterials (CK). The error bars indicate the standard deviations of three replications.

**FIGURE 5:**
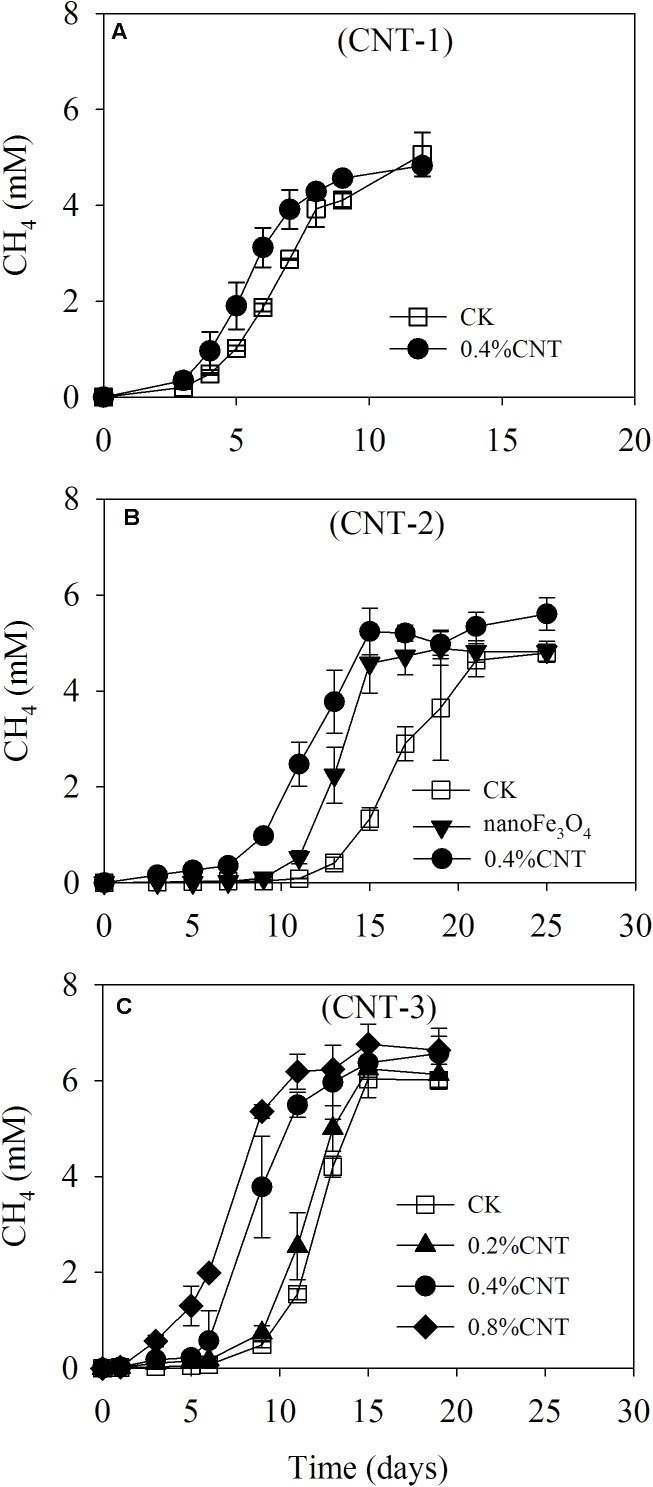
Methane production from butyrate oxidation in the enriched incubation in the presence of carbon nanotubes (CNTs). Three continuous transfers were made. The first transfer **(A)** was inoculated from the 13th transfer of the nanoFe_3_O_4_-amended enrichment with the addition of CNTs (0.4% CNT). The second transfer **(B)** was made by inoculating the first CNT-amended transfer into the fresh media with the addition of nanoFe_3_O_4_ (nanoFe_3_O_4_) and CNTs (0.4% CNT). The third transfer **(C)** was prepared by inoculating the second CNT-amended transfer into fresh media of different CNT concentrations, i.e., 0.2, 0.4, and 0.8% (w/v). A control without CNTs (CK) was prepared in parallel for every transfer. The error bars indicate the standard deviations of three replications.

Various tests were carried out on the enriched cultivation (**Figure 4**). Addition of nanoFe_3_O_4_ markedly stimulated the production of CH_4_ as already observed in the transfer incubations. However, silica coating of nanoFe_3_O_4_ completely eliminated this stimulatory effect. On the other hand, the addition of graphite retained the stimulatory effect albeit less significant compared with nanoFe_3_O_4_. For the CNTs test, three further transfers were performed (**Figure 5**). A slight positive effect was detected in the first CNTs transfer (**Figure 5A**). In the second transfer, the first CNTs enrichment was re-inoculated to fresh media in the presence of CNTs or nanoFe_3_O_4_. Significant positive effects were observed for both CNTs and nanoFe_3_O_4_ (**Figure 5B**). In the third CNTs transfer, the effect of CNTs concentrations was determined. The stimulatory effect was substantiated at the concentration of 0.4 and 0.8% CNTs (w/v) but diminished when the concentration decreased to 0.2% CNTs.

**FIGURE 6:**
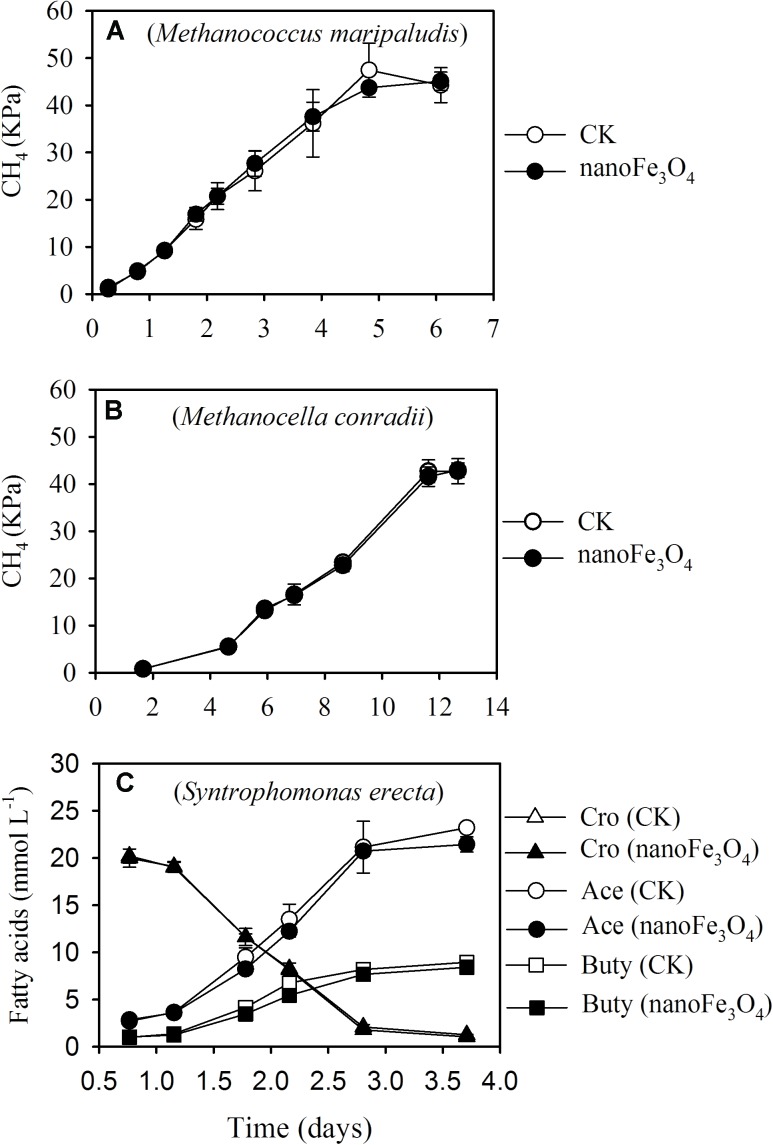
Effect of nanoFe_3_O_4_ on CH_4_ production by two hydrogenotrophic methanogens, *Methanococcus maripaludis* **(A)** and *Methanocella conradii* **(B)**, and on crotonate fermentation by a butyrate syntroph *Syntrophomonas erecta* **(C)** under axenic conditions. CK, the control without nanoFe_3_O_4_. For crotonate fermentation, the concentrations of crotonate (Cro), acetate (Ace), and butyrate (Buty) were monitored. The error bars indicate the standard deviations of three replications.

Three pure culture strains were used to test the effect of nanoFe_3_O_4_. Incubation of *Methanococcus maripaludis* (**Figure 6A**) and *Methanocella conradii* (**Figure 6B**) with 170 kPa of H_2_ produced 43–45 kPa of CH_4_, indicating the stoichiometric production of CH_4_ from CO_2_ reduction by H_2_. Addition of nanoFe_3_O_4_ did not show an effect on CH_4_ production by these two methanogen strains. Incubation of *Syntrophomonas erecta* (**Figure 6C**) with 20 mM crotonate yielded approximately 22 mM acetate and 8.7 mM butyrate, thus more than a half of crotonate was oxidized to acetate and the remaining was reduced to butyrate. Similar to CH_4_ production, addition of nanoFe_3_O_4_ did not reveal an effect on crotonate fermentation in pure culture.

## Discussion

A highly enriched syntrophic consortium was obtained from the wetland sediment after enrichment incubation with continuous transfers in the presence of nanoFe_3_O_4_. Mass balance and isotopic labeling indicated that the conversion of butyrate in the enrichment followed the stoichiometric reduction of CO_2_ by electrons released from butyrate oxidation. Molecular analyses revealed that *Syntrophomonas* closely related to a *Syntrophomonas wolfei* strain were left as the only bacteria and methanogens were dominated exclusively by *Methanobacteria* in addition to a few *Methanocella* in the enrichment. The aceticlastic methanogens existing initially was possibly lost after a few continuous transfers and *Geobacter* were not detected. Thus, it can be assumed that butyrate oxidation and CH_4_ production resulted solely from the syntrophic interaction between *Syntrophomonas* and *Methanobacteria* together with a few *Methanocella* in the enrichment.

The addition of nanoFe_3_O_4_ consistently shortened the lag period and enhanced the maximum rate of CH_4_ production compared with the control without nanoFe_3_O_4_. This is consistent with our previous observation that nanoFe_3_O_4_ promoted syntrophic oxidation of butyrate in paddy field soil and lake sediment enrichments (Li et al., 2015; Zhang and Lu, 2016). A few other studies showed that nanoFe_3_O_4_ promoted syntrophic oxidation of ethanol and propionate in paddy soil and anaerobic bioreactors (Kato et al., 2012a; Viggi et al., 2014; Jing et al., 2017). But microbial compositions in previous enrichments were more complex and often *Geobacter* species were present. Due to the complicated microbial compositions, it was difficult to pinpoint explicitly the microbial interaction and figure out where the stimulatory effect of nanoFe_3_O_4_ was located. The enrichment obtained in the present study, however, was highly enriched without the presence of *Geobacter* and other bacteria. It can be concluded that the stimulatory effect of nanoFe_3_O_4_ is directly due to the response of *Syntrophomonas* and *Methanobacteria*/*Methanocella*.

Different mechanisms may be involved in the stimulatory effect of nanoFe_3_O_4_. Firstly, nanoFe_3_O_4_ has a relatively low redox potential (Straub et al., 2001). It has been argued that the stimulatory effect by nanomaterials like CNTs on syntrophic coculture and pure culture of methanogens can be due to the decrease in redox potential (Salvador et al., 2017). Similar effect may be postulated for nanoFe_3_O_4_. Therefore, we tested its effect on three pure culture strains. The production of CH_4_ by two hydrogenotrophic methanogens and the fermentation of crotonate by a *Syntrophomonas* strain revealed no effect by nanoFe_3_O_4_. Though not the identical representatives of syntrophs and methanogens in the enrichment, the effect of nanoFe_3_O_4_ on individual organisms by reducing the redox potential might be excluded in the present study. Secondly, the physical support of nanoparticles for microbial aggregate formation through adsorption of microbial cells may also result in a stimulatory effect on syntrophy. Accordingly, we modified nanoFe_3_O_4_ with silica coating which insulated the electric conductivity of nanoFe_3_O_4_ but otherwise retained the physical support for microbial aggregation. Silica coating, however, completely eliminated the stimulatory effect of nanoFe_3_O_4_, in consistence with previous study (Li et al., 2015). More tests were then conducted concerning the effect of electric conductivity. We substituted nanoFe_3_O_4_ with CNTs and graphite in the enriched consortium. Three transfers were made using CNTs, which all showed the stimulatory effect. Moreover, the effect appeared increasing with the concentration of CNTs in the medium, and re-inoculation of CNT-amended enrichment into nanoFe_3_O_4_ medium did not influence the later effect. Application of graphite also showed the enhancement of CH_4_ production compared with the control, consistent with previous study (Li et al., 2015). Apart from the common property in electric conductivity, nanoFe_3_O_4_, CNTs and graphite are different chemically and physically. Together, the results from above tests suggest that the electric conductivity of nanomaterials plays the key role in promoting the syntrophic oxidation of butyrate.

*Geobacter* species have e-pili and outer membrane *c*-type cytochromes. Some of *Methanosarcina* and *Methanosaeta* species have been demonstrated to perform DIET with *Geobacter* (Morita et al., 2011; Liu et al., 2012; Rotaru et al., 2014a,b). Recently, it was proposed that DIET also occurred in the anaerobic methanotrophic consortia consisting of ANME-2 with the putative mechanism linked to the presence of large multi-heme cytochromes (McGlynn et al., 2015; Wegener et al., 2015). However, *Syntrophomonas* species do not contain genomic inventories coding for conductive e-pili and outer membrane cytochromes (Sieber et al., 2012) and unlike *Methanosarcina*, *Methanosaeta*, and ANME-2, the *Methanobacteria* represent methanogens without cytochromes (Thauer et al., 2008). Therefore, it appears hard to conceive that DIET occurs between *Syntrophomonas* and *Methanobacteria*. But the essentiality of biological electric connection has been challenged in the experiments using *Geobacter* mutants (Liu et al., 2012; Rotaru et al., 2014a; Liu et al., 2015). Supplementation of conductive granular activated carbon and magnetite nanoparticles restored DIET in mutants depleted of biological electric connections. Before a better alternate explanation can be uncovered for the stimulatory effect of nanomaterials observed in the present study, we assume that DIET is induced for butyrate oxidation by the biologically compatible conductive nanomaterials. Recently, the membrane associated (reverse) electron transfer chain in *Syntrophomonas wolfei* has been proposed (Sieber et al., 2015) and the surface-oriented hydrogenases and formate dehydrogenases were abundant in both *Syntrophomonas* and hydrogenotrophic methanogens (Thauer et al., 2008; Sieber et al., 2012; Worm et al., 2014). It warrants further investigations whether these components can be involved in DIET in concert with H_2_/formate transfer for the syntrophic oxidation of butyrate.

## Conclusion

A highly enriched consortium comprising *Syntrophomonas* and *Methanobacteria*-*Methanocella* was obtained from Tibetan Plateau wetland sediment. The syntrophic production of CH_4_ from butyrate oxidation was substantially accelerated in the presence of nanoFe_3_O_4_. We propose that DIET is likely induced by the added conductive materials in butyrate syntrophy. Mechanisms different from those in *Geobacter* species may operate in syntrophic butyrate oxidation and shall deserve further investigations. The conductive minerals like magnetite and pyrite are ubiquitous in soils and sediments. Further investigations shall also pay an attention to the effect of these materials on the anaerobic decomposition of organic substances and methanogenesis in those habitats.

## Author Contributions

YL conceived the research. LF performed the enrichment cultivation, isotope labeling, and molecular analysis. TS and JZ tested the effect of CNTs on the enrichment. WZ performed the pure culture test. YL wrote the manuscript. YL and LF edited the manuscript. All authors reviewed and approved the manuscript.

## Conflict of Interest Statement

The authors declare that the research was conducted in the absence of any commercial or financial relationships that could be construed as a potential conflict of interest.
