# Supplementary figures and images for: Stimulatory Effect of Magnetite Nanoparticles on a Highly Enriched Butyrate-Oxidizing Consortium
Source: Front Microbiol. 2018 Jul 5;9:1480. doi: 10.3389/fmicb.2018.01480 (PMC6041394; doi:10.3389/fmicb.2018.01480)

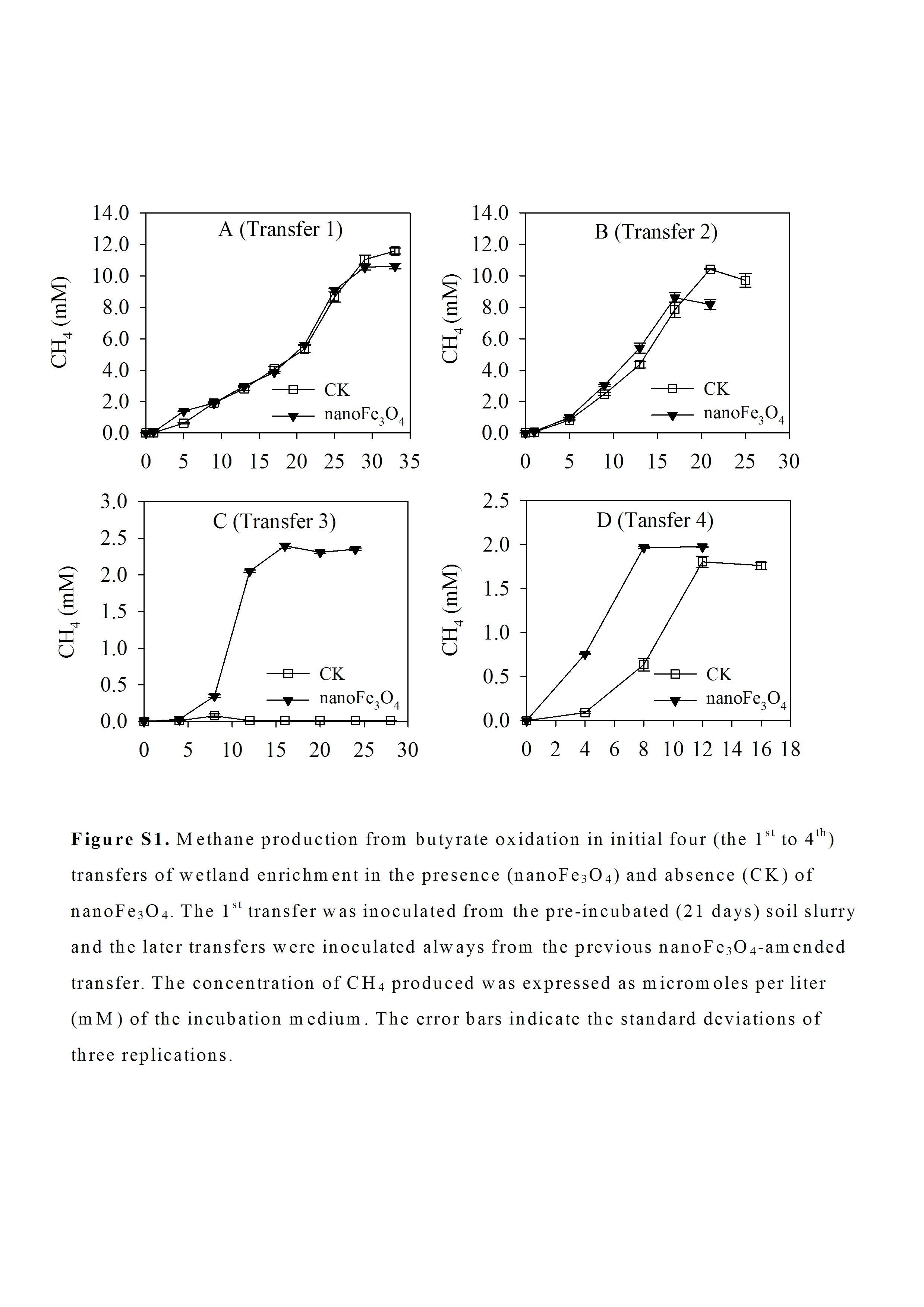

Supplement: Supplementary file 1 [file Image_1.TIF]

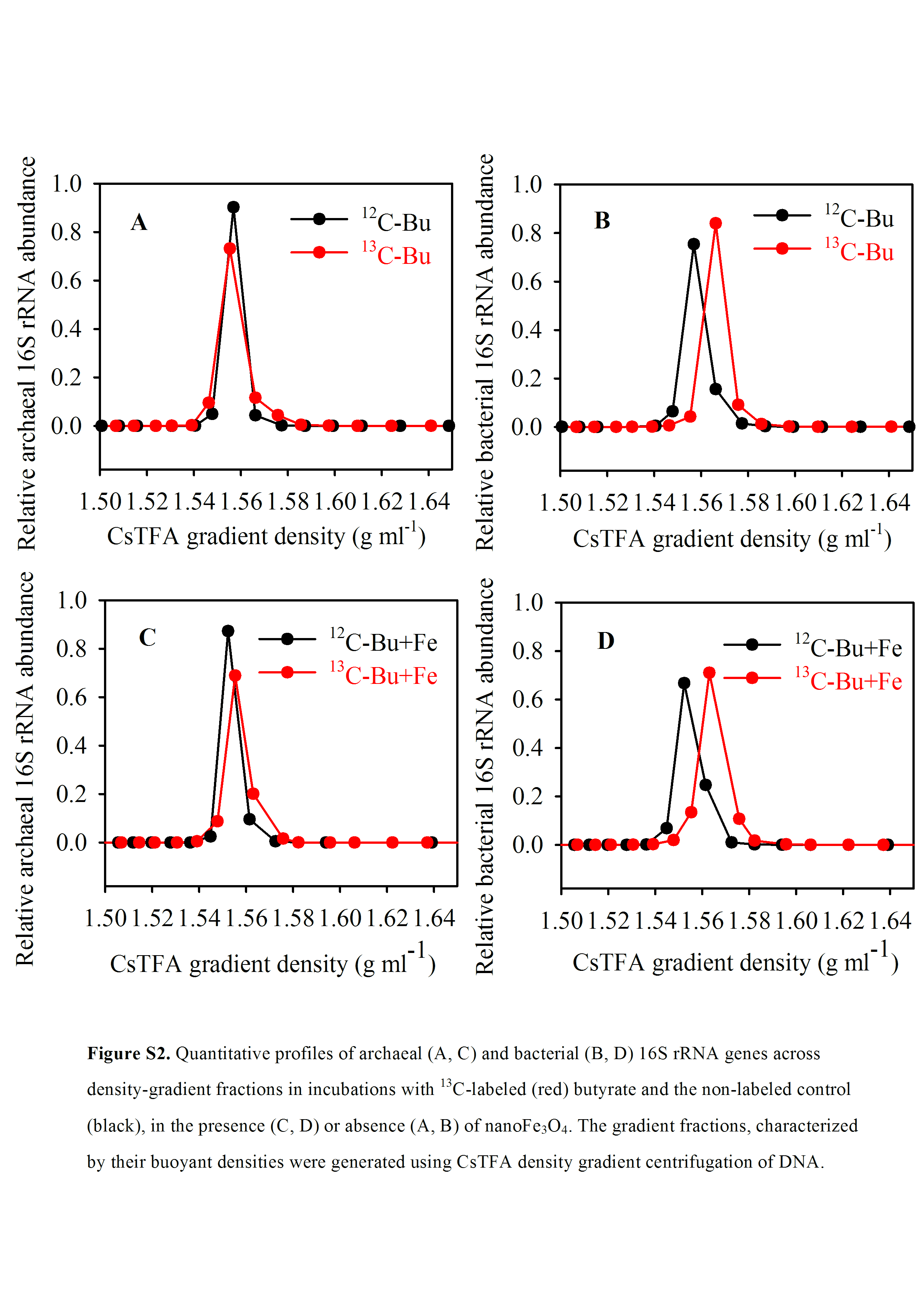

Supplement: Supplementary file 2 [file Image_2.TIF]

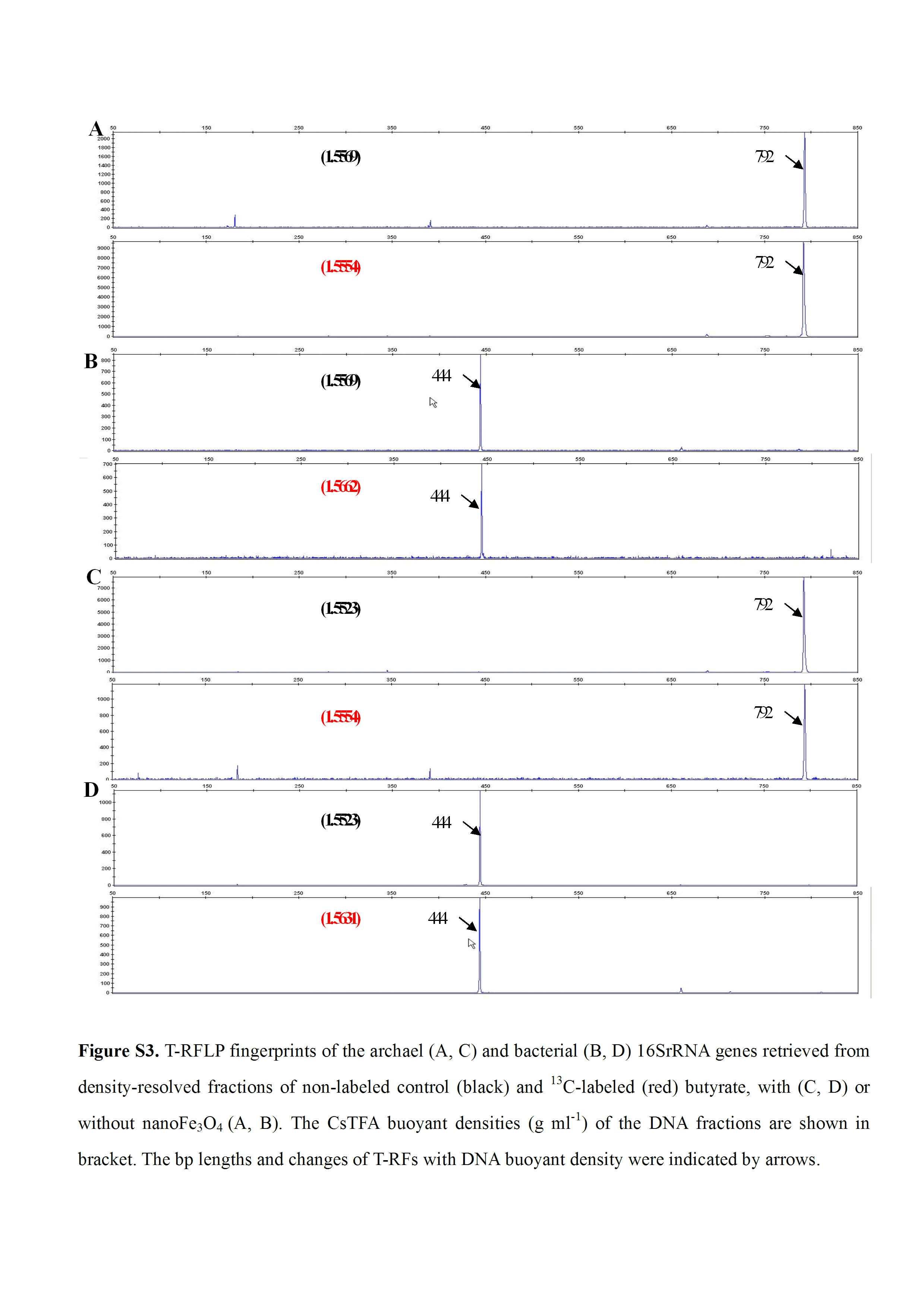

Supplement: Supplementary file 3 [file Image_3.TIF]
